# Age-related changes in brain signal variability in autism spectrum disorder

**DOI:** 10.1186/s13229-024-00631-3

**Published:** 2025-02-08

**Authors:** Priyanka Sigar, Nicholas Kathrein, Elijah Gragas, Lauren Kupis, Lucina Q. Uddin, Jason S. Nomi

**Affiliations:** 1 Department of Psychiatry and Biobehavioral Sciences, Semel Institute for Neuroscience and Human Behavior, University of California Los Angeles, Los Angeles, CA 90024 USA; 2 Department of Psychology, University of California Los Angeles, Los Angeles, CA 90024 USA

**Keywords:** Age, ASD, Brain–behavior relationships, Resting-state fMRI, Mean square successive difference

## Abstract

**Background:**

Brain signal variability (BSV) is an important understudied aspect of brain function linked to cognitive flexibility and adaptive behavior. Autism spectrum disorder (ASD) is a neurodevelopmental condition characterized by social communication difficulties and restricted and repetitive behaviors (RRBs). While atypical brain function has been identified in individuals with ASD using fMRI task-activation and functional connectivity approaches, little is known about age-related relationships with resting-state BSV and repetitive behaviors in ASD.

**Methods:**

We conducted a cross-sectional examination of resting-state BSV and its relationship with age and RRBs in a cohort of individuals with Autism Brain Imaging Data Exchange (n = 351) and typically developing (TD) individuals (n = 402) aged 5–50 years obtained from the Autism Brain Imaging Data Exchange. RRBs were assessed using the Autism Diagnostic Interview-Revised (ADI-RRB) scale. BSV was quantified using the root-mean-square successive difference (rMSSD) of the resting-state fMRI time series. We examined categorical group differences in rMSSD between ASD and TD groups, controlling for both linear and quadratic age. To identify dimensional relationships between age, group, and rMSSD, we utilized interaction regressors for group x age and group x quadratic age. Within a subset of individuals with ASD (269 subjects), we explored the relationship between rMSSD and ADI-RRB scores, both with and without age considerations. The relationship between rMSSD and ADI-RRB scores was further analyzed while accounting for linear and quadratic age. Additionally, we investigated the relationship between BSV, age, and ADI-RRB scores using interaction regressors for age x RRB and quadratic age x RRB.

**Results:**

When controlling for linear age effects, we observed significant group differences between individuals with ASD and TD individuals in the default-mode network (DMN) and visual network, with decreased BSV in ASD. Similarly, controlling for quadratic age effects revealed significant group differences in the DMN and visual network. In both cases, individuals with ASD showed decreased BSV compared with TD individuals in these brain regions. The group × age interaction demonstrated significant group differences in the DMN, and visual network brain areas, indicating that rMSSD was greater in older individuals compared with younger individuals in the ASD group, while rMSSD was greater in younger individuals compared with older individuals in the TD group. The group × quadratic age interaction showed significant differences in the brain regions included in DMN, with an inverted U-shaped rMSSD-age relationship in ASD (higher rMSSD in younger individuals that slightly increased into middle age before decreasing) and a U-shaped rMSSD-age relationship in TD (higher rMSSD in younger and older individuals compared with middle-aged individuals). When controlling for linear and quadratic age effects, we found a significant positive association between rMSSD and ADI-RRB scores in brain regions within the DMN, salience, and visual network. While no significant results were observed for the linear age × RRB interaction, a significant association between quadratic age and ADI-RRB scores emerged in the DMN, dorsal attention network, and sensorimotor network. Individuals with high ADI-RRB scores exhibited an inverted U-shaped relationship between rMSSD and age, with lower rMSSD levels observed in both younger and older individuals, and higher rMSSD in middle-aged individuals. Those with mid-range ADI-RRB scores displayed a weak inverted U-shaped rMSSD-age association. In contrast, individuals with low ADI-RRB scores showed a U-shaped rMSSD-age association, with higher rMSSD levels in younger and older individuals, but a lower rMSSD in middle-aged individuals.

**Conclusion:**

These findings highlight age-related atypical BSV patterns in ASD and their association with repetitive behaviors, contributing to the growing literature on understanding alterations in functional brain maturation in ASD.

**Supplementary Information:**

The online version contains supplementary material available at 10.1186/s13229-024-00631-3.

## Introduction

Once regarded as noise, brain signal variability (BSV) has emerged as a crucial component of optimal brain function [1]. For instance, the 'Bayes optimal theory' suggests that a neuronal population chooses an appropriate response to a specific stimulus from a wide range of potential responses. This response variability enables flexible neural firing, which in turn allows neural networks to adapt effectively to different circumstances [2]. Previous fMRI research has shown that BSV is associated with task performance [3], lifespan stage [4], and clinical symptoms in neurodevelopmental disorders [5]. However, little work has examined how BSV is related to age and behavioral symptom severity in autism spectrum disorder (ASD) using large samples.

Recently, the analysis of BSV has emerged as a valuable tool for understanding age-related functional brain differences. Using fMRI, [6] one study found that during fixation periods of a task, the standard deviation (SD) of the blood-oxygen level-dependent (BOLD) time series was higher in younger adults compared with older adults. It has also been shown that BOLD SD is higher in individuals who perform better on cognitive tasks [7], is sensitive to parametric modulations in task difficulty [8], is sensitive to amphetamine administration during task performance [9], and is higher for external compared with internal or intrinsic states of cognitive processing [10]. BSV has also been investigated using the mean square successive difference (MSSD) of the BOLD signal and has been shown to index financial risk-taking across age [11]. BSV computed with MSSD shows reductions across most brain regions over the lifespan, with age-related increases noted only within the insula and ventral temporal cortex [4]. Our group recently found that associations between BSV and executive function change as a function of age [12]. These studies demonstrate how the analysis of BSV can offer a valuable perspective on the links between brain function, cognitive function, and age.

While several studies have explored the association between BSV and neurodevelopmental [13], and psychiatric disorders [14], only one prior study has examined BSV in children and adolescents with and without ASD [5]. Using a categorical analysis on 20 subjects with ASD and 17 typically developing subjects, they found no categorical group differences in MSSD between children and adolescents (aged 8–18 years old) with and without an ASD diagnosis. However, a dimensional approach revealed positive correlations between BSV and age in all participants, and negative correlations between BSV and the severity of ASD behaviors (repetitive behaviors, social responsiveness) in frontal, parietal, and occipital brain areas among others. This initial study demonstrates how BSV may be used to characterize symptom severity in individuals with ASD. However, no study has yet investigated the relationships between BSV, age, and repetitive behaviors in ASD using the large sample sizes available from the Autism Brain Imaging Data Exchange (ABIDE) I and II databases. Examining age-related BSV patterns and how they are associated with RRBs in ASD within such a large sample size will help to provide insights into these important developmental relationships.

The current study used resting-state fMRI scans from the ABIDE I and II databases [5, 6] to conduct two main analyses to examine developmental patterns of BSV in individuals with ASD and TD individuals. The first analysis examined how BSV (quantified using the root mean square MSSD (rMSSD)) may differentiate between individuals with ASD (n = 351) and TD individuals (n = 402) (5 to 50 years of age) with and without considering the influence of age. The second analysis examined how BSV is related to RRB symptom severity (measured using the ADI-RRB scale) across development with and without considering the influence of age on a subset of individuals with ASD (9 sites; 269 subjects). Based on previous literature examining BSV in neurodevelopmental [13] and psychiatric disorders [14, 15], we expected to observe age-related atypical BSV in ASD. Additionally, we anticipated that age would play a critical role in modulating the relationship between BSV and repetitive behavior in individuals with ASD.

## Methods

### Participants

Publicly available resting-state fMRI data was downloaded from the ABIDE database (ABIDE-I, ABIDE-II; https://fcon_1000.projects.nitrc.org/indi/abide); [16, 17] consisting of 753 participants (351 ASD; 308 male/43 female) and 402 TD; 316 male/86 female) across nine sites—Eidgenössische Technische Hochschule Zurich (ETH), Georgetown University (GU), New York University (NYU) Langone Medical Center, San Diego State University (SDSU), Trinity Center for Health Sciences (TCD), University of California Davis (UCD), University of Michigan (UM), University of Utah School of Medicine (USM), and Yale Child Study Center (Yale). To minimize between-site differences, we selected sites that used an eyes-open resting-state protocol (at ETH, NYU, SDSU, TCD, and UM, subjects were required to focus on a fixation cross, while subjects at the other sites were instructed to mind-wander). Additionally, all sites used a TR of 2 s (Table 1; subject IDs are listed in Additional file 2). This criterion is crucial as rMSSD is influenced by the acquisition rate; higher TRs yield larger rMSSD values due to the increased time between successive BOLD signal volumes [4]. ASD and TD groups were matched for age, MRI head motion, and IQ within each site (*p-value* > 0.07). During preprocessing, all datasets were standardized to either 6 min or 5 min, depending on the scan run time. If the scan run time exceeded 6 min, it was truncated to 6 min. If the scan run time was less than 6 min, it was truncated to 5 min. The first five volumes (10 s) were removed to allow the MRI signal to reach equilibrium. Out of the nine sites, six (ETH, NYU, SDSU, UM, USM, Yale) had a resting-state scan time of six minutes, while the remaining three (GU, Trinity, UCD) had a scan time of five minutes. Data collection and sharing procedures were approved by the review board at each institution. Written informed consent and/or informed assent were obtained from each participant and their parents, where applicable. Individuals with poor functional or structural scans or average framewise displacement (FD) > 0.5 mm were eliminated from the analysis [18].

**Table 1:** Demographics

Site	ASD (n)	TD (n)	Age Range (mean ± SD)		*p*^a^	Average FD Range (mean ± SD)		*p*^a^
			ASD	TD		ASD	TD	
*ABIDE I*								
NYU	63	86	7–39 (15 ± 7)	6–31 (15 ± 5)	*0.64*	0.06–0.23 (0.13 ± 0.04)	0.05–0.31 (0.12 ± 0.05)	*0.27*
SDSU	12	22	12–17 (15 ± 1)	8–16 (14 ± 1)	*0.21*	0.04–0.22 (0.11 ± 0.06)	0.06–0.25 (0.13 ± 0.06)	*0.29*
UM	50	67	9–18 (13 ± 2)	8–28 (14 ± 3)	*0.11*	0.07–0.39 (0.18 ± 0.08)	0.08–0.41 (0.17 ± 0.08)	*0.35*
USM	41	35	11–50 (23 ± 8)	9–39 (21 ± 7)	*0.41*	0.05–0.30 (0.17 ± 0.07)	0.06–0.35 (0.16 ± 0.06)	*0.44*
TCD	23	24	12–25 (17 ± 3)	12–25 (17 ± 3)	*0.84*	0.12–0.33 (0.20 ± 0.06)	0.11–0.40 (0.17 ± 0.06)	*0.07*
YALE	25	27	7–17 (13 ± 3)	8–17 (12 ± 2)	*0.81*	0.07–0.27 (0.17 ± 0.06)	0.06–0.35 (0.15 ± 0.07)	*0.35*
*ABIDE II*								
ETH	8	23	14–27 (21 ± 3)	13–30 (23 ± 4)	*0.14*	0.10–0.45 (0.22 ± 0.12)	0.08–0.27 (0.17 ± 0.05)	*0.15*
GU	42	51	8–13 (11 ± 1)	8–13 (10 ± 1)	*0.07*	0.06–0.45 (0.21 ± 0.09)	0.07–0.44 (0.18 ± 0.08)	*0.12*
NYU	33	19	5–34 (10 ± 6)	5–12 (8 ± 2)	*0.39*	0.09–0.29 (0.17 ± 0.06)	0.09–0.38 (0.18 ± 0.08)	*0.77*
SDSU	32	25	7–18 (13 ± 3)	8–17 (13 ± 3)	*0.82*	0.04–0.41 (0.12 ± 0.08)	0.06–0.36 (0.14 ± 0.07)	*0.31*
UCD	15	14	12–17 (14 ± 1)	12–17 (14 ± 1)	*0.63*	0.07–0.37 (0.17 ± 0.08)	0.08–0.35 (0.16 ± 0.09)	*0.76*
USM	7	9	9–38 (21 ± 9)	11–36 (22 ± 8)	*0.84*	0.09–0.42 (0.22 ± 0.11)	0.06–0.21 (0.13 ± 0.05)	*0.40*
Total	351	402	5–50					

^a^Two-sample t-test, *SD* Standard deviation, *ETH* Eidgenössische Technische Hochschule, *GU* Georgetown University, *NYU* New York University Langone Medical Center, *SDSU* San Diego State University, *TCD* Trinity Center for Health Sciences, *UCD* University of California Davis, *UM* University of Michigan, *USM* University of Utah School of Medicine

At each site, inclusion as an individual with ASD required a diagnosis by a clinician using the following criteria: Diagnostic and Statistical Manual of Mental Disorders (DSM) versions four text revision (DSM-IV-TR) and five (DSM-V); Autism Diagnostic Observation Schedule (ADOS) general (ADOS-G) or version two (ADOS-2); Autism Diagnostic Interview-Revised (ADI-R). Each site conducted separate procedures concerning which criteria were administered. A total of 66 participants diagnosed with ASD were using psychotropic medications.

*Data Preprocessing.* Resting-state fMRI data were preprocessed as in our previous BSV study [4] using the Data Processing Assistant for Resting-State fMRI toolbox (DPARSF advanced edition v4.1_160415, http://rfmri.org/DPARSF) [19], along with FSL and AFNI functions. The initial five volumes were discarded to ensure steady signal stabilization. On the remaining images, we applied despiking (AFNI’s 3dDespike), realignment (DPARSF-A), normalization to MNI space (DPARSF-A), and cluster-based smoothing (AFNI’s 3dBlurToFWHM).

An ICA-FIX classifier [20] was individually trained for each site. For the GU, NYU, and USM sites, 20 subjects (comprising 10 TD and 10 ASD) were used for training [4, 21]. For the SDSU and UM sites, 18 subjects (9 TD and 9 ASD each) were used. For the ETH, Trinity, UCD, and Yale sites we used 16 subjects (8 TD and 8 ASD) at each site for training. These subjects were randomly chosen across a range of low to high head motion and across younger and older individuals. Noise components consisting of white matter, scanner noise, head motion, cardiac, and respiration artifacts were identified using visual inspection of ICA spatial maps, power frequency spectra, and time-series appearance. The classifications were then fed into ICA-FIX and used to identify and regress noise components from the remaining subjects within each site. Prior studies have demonstrated that ICA denoising effectively eliminates non-neuronal sources of variability while increasing effect sizes in variability analyses [6], and effectively mitigates cross-site differences in fMRI analyses [22]. Finally, 24 motion parameters (6 head motion parameters, 6 head motion parameters one time point before, and the 12 corresponding squared items) were regressed from the data before linear detrending and band-pass filtering (0.01–0.1 Hz) (DPARSF-A). To address scanner differences, we applied ComBat harmonization using a MATLAB-based implementation of the NeuroCombat [23] to correct for effects on the BSV measure across different scanner sites while preserving biological variability.

*Calculation of Voxel-wise rMSSD:* BOLD signal variability was estimated using rMSSD, which considers the temporal continuity of resting-state fMRI time-series and quantifies the average distance in signal amplitude between successive time points [24]. Time-series were normalized to z-statistics before calculating voxel-wise rMSSD for each subject using custom MATLAB scripts [4, 11]. rMSSD was calculated for each time series by subtracting the value at time point t + 1 from the value at time point t, squaring the result to account for negative values, then calculating the square root of the average of all differences [24]. Associations between rMSSD, age and RRB scores were calculated in FSL using ordinary least squares (OLS) regression with FD was included as a nuisance regressor.

### rMSSD differences between ASD and TD

All analyses were conducted using subject-level whole-brain voxel-wise rMSSD spatial maps as the dependent variable (DV) using OLS regression in FSL. Categorical group differences in rMSSD between ASD and TD groups were assessed controlling for linear (Model 1) and quadratic age (Model 2). Dimensional relationships between age, group, and rMSSD were identified using group × age (Model 3) and group × quadratic age (Model 4) interaction regressors. There was no significant correlation between FD and age in the ASD group (*rho* = *− 0.09, p* = *0.06*), and a weak negative relationship in the TD group (*rho* = *− 0.15, p* = *0.002*). Head motion was accounted for as a nuisance regressor in all analyses. All regression models used both liberal and stringent thresholds (z > 2.3 and 3.3, voxel-wise uncorrected) to ensure a balance between spatial specificity, sensitivity, and repeatability [25]. Additionally, cluster-wise correction was applied using Gaussian Random Field (GRF) theory (*p* < 0.05, corrected) [26].

## Model 1:

$$rMSSD= \beta 0 + \beta 1\left(Group\right)+ \beta \left(Age\right) + \beta 3\left(FD\right) + \epsilon $$

## Model 2:

$$rMSSD=\, \beta 0 + \beta 1\left(Group\right) + \beta 2\left(Age\right) + \beta 3\left(Quadratic \,Age\right) + \beta 4\left(FD\right) + \epsilon $$

## Model 3:

$$rMSSD=\, \beta 0 + \beta 1\left(Group\right) + \beta 2\left(Age\right) + \beta 3\left(Group \times Age\right) + \beta 4\left(FD\right) + \epsilon $$

## Model 4:

$$rMSSD=\, \beta 0 + \beta 1\left(Group\right) + \beta 2 \left(Age\right) + \beta 3\left(Quadratic\, Age\right) + \beta 4\left(Group \times Age\right)+ \beta 5\left(Group \times Quadratic\, Age\right) + \beta 6\left(FD\right) + \epsilon $$

### rMSSD and ADI-RRB scores in ASD

The relationship between rMSSD and ADI-RRB scores was explored within a subset of individuals with ASD (9 sites; 269 subjects) with and without the consideration of age. The relationship between rMSSD and ADI-RRB scores was examined while controlling for age (Model 5) and quadratic age (Model 6). The relationship between BSV, age, and ADI-RRB scores was examined using the age x RRB (Model 7) and the quadratic age x RRB (Model 8) interaction regressors. There was no significant correlation between FD and ADI-RRB scores (*rho* = *0.01, p* = *0.7*). FD was included as a nuisance regressor in all models. The scatterplots signifying the age × RRB interaction categorized the subjects into three groups based on their ADI-RRB scores (low, mid, high) using equal binning intervals for visualization purposes only (Fig. 3D).

## Model 5:

$$rMSSD=\, \beta 0 + \beta 1\left(Group\right) + \beta 2\left(Age\right) + \beta 3\left(RRB\right) + \beta 4\left(FD\right) + \epsilon $$

## Model 6:

$$rMSSD=\, \beta 0 + \beta 1\left(Group\right) + \beta 2\left(Age\right) + \beta 3\left(Quadratic\, Age\right) + \beta 4\left(RRB\right)+ \beta 5\left(FD\right)+ \epsilon $$

## Model 7:

$$rMSSD=\, \beta 0 + \beta 1\left(Group\right) + \beta 2\left(Age\right) + \beta 3\left(RRB\right) + \beta 4\left(Age \times RRB\right)+ \beta 5\left(FD\right)+ \epsilon $$

## Model 8:

$$rMSSD=\, \beta 0 + \beta 1\left(Group\right) + \beta 2\left(Age\right) + \beta 3\left(Quadratic\, Age\right) + \beta 4\left(RRB\right)+ \beta 5\left(Age \times RRB\right)+ \beta 6\left(Quadratic\, Age \times RRB\right)+ \beta 5\left(FD\right)+ \epsilon $$

### Reproducibility analysis

To examine how IQ, gender, and handedness may influence the results, post-hoc analyses were conducted on a subset of individuals with available demographics as nuisance covariates across models 1–4 (ASD: n = 336; TD: n = 389) and across models 5–8 (ASD: n = 256) to reproduce significant effects found in the main analysis. Demographics for IQ and gender are available in Additional Table 1.

## Results

### rMSSD differences between ASD and TD

**Fig. 1:**
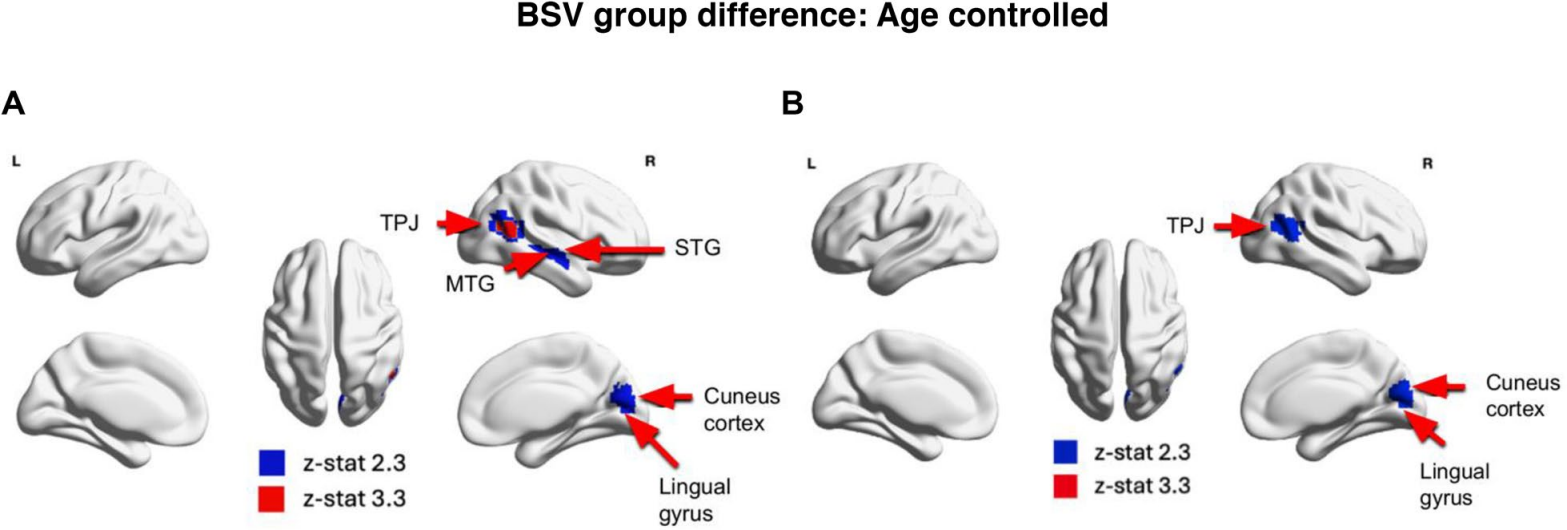
**A** Results from regression Model 1 where individuals with ASD show significantly lower rMSSD than TD individuals when controlling for age (*p* < 0.05 cluster-wise corrected). **B** Results from regression Model 2 where individuals with ASD show significantly lower rMSSD than TD individuals when controlling for quadratic age (*p* < 0.05 cluster-wise corrected). *rMSSD* root mean-square successive difference, *TD* typically developing, *ASD* autism spectrum disorder, *TPJ* temporal-parietal junction, *STG* superior temporal gyrus, *MTG* medial temporal gyrus

When controlling for linear age effects (Model 1), we observed significant group differences between individuals with ASD and TD individuals in the temporal-parietal junction (TPJ) (voxel-wise uncorrected threshold (z > 3.3)) and middle temporal gyrus (MTG), superior temporal gyrus (STG), cuneus cortex, and lingual gyrus (voxel-wise uncorrected thresholds (z > 2.3)) (Fig. 1A).

When controlling for quadratic age effects (Model 2), there were similar significant group differences in the TPJ, cuneus cortex and lingual gyrus. However, these differences were observed at a more lenient voxel-wise uncorrected threshold (z > 2.3) (Fig. 1B). In both cases, individuals with ASD exhibited decreased BSV compared with TD individuals in these brain regions.

**Fig. 2:**
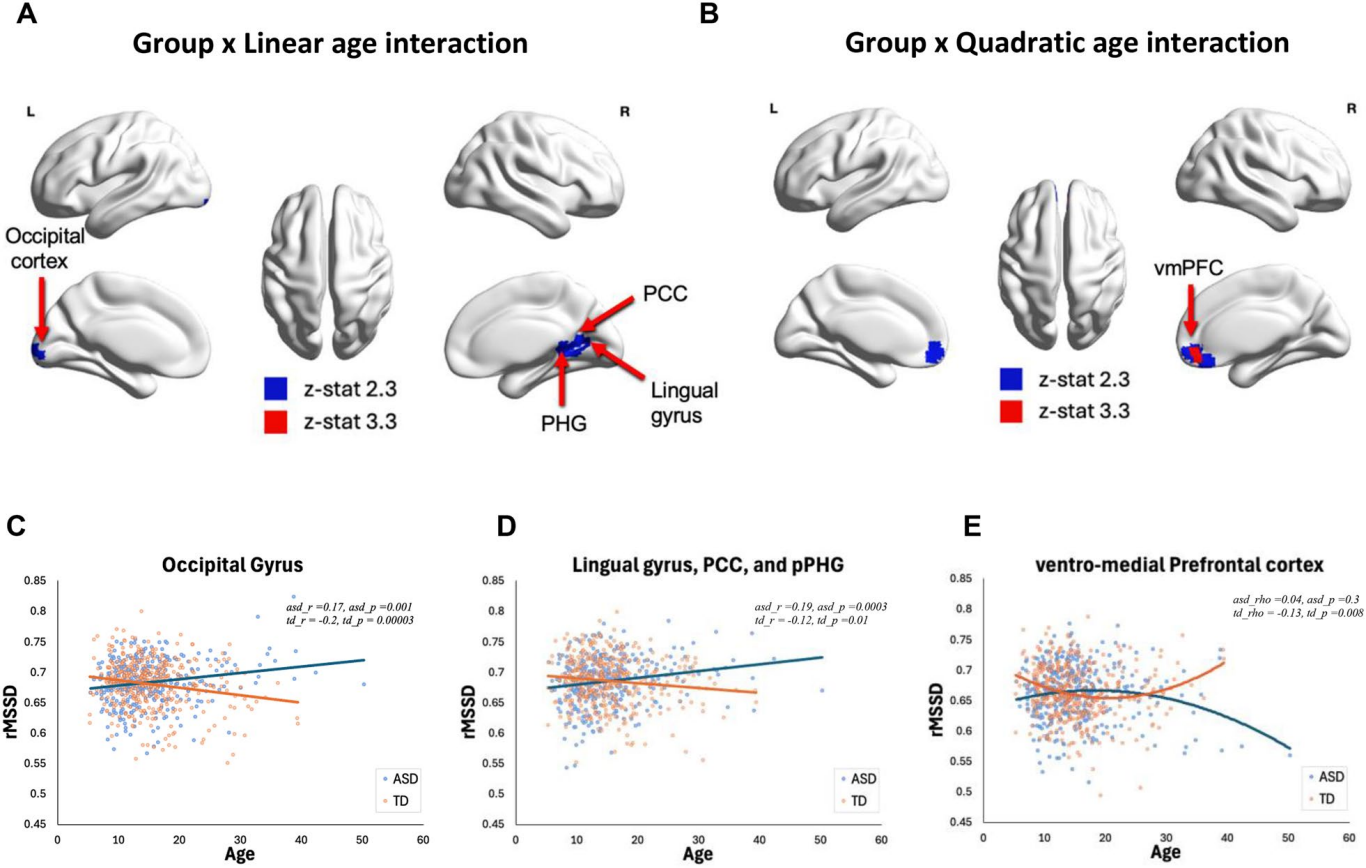
**A** Results from regression Model 3 where a Group x Linear age interaction shows a positive linear relationship between rMSSD and age for individuals with ASD while TD individuals show a negative linear relationship between rMSSD and age in the PCC, PHG, lingual gyrus, and occipital cortex (**C**, **D**). **B** Results from regression Model 4 where a Group x Quadratic age interaction shows an inverted U-shaped relationship between rMSSD and age for individuals with ASD while TD individuals show a U-shaped relationship between rMSSD and age in the medial prefrontal cortex (**E**). *rMSSD* root mean-square successive difference, *TD* typically developing, *ASD* autism spectrum disorder, *PCC* posterior cingulate cortex, *pPHG* posterior Para-hippocampal gyrus, *vmPFC* ventro-medial prefrontal cortex

The group x age interaction (Model 3) showed significant group differences in the posterior cingulate cortex (PCC), parahippocampal gyrus (PHG), lingual gyrus, and occipital pole (z > 2.3 voxel-wise uncorrected). These interactions showed that rMSSD was greater in older individuals compared with younger individuals in the ASD group, while rMSSD was greater in younger individuals compared with older individuals in the TD group (Fig. 2C, D).

The group x quadratic age interaction (Model 4) showed significant group differences in the medial prefrontal cortex (mPFC) (z > 3.3 voxel-wise uncorrected) (Fig. 2B). These interactions showed that the ASD group had an inverted U-shaped rMSSD-age relationship where younger individuals had low rMSSD that increased slightly into middle age before dropping down in older age, while the TD group had a U-shaped rMSSD-age relationship where younger and older individual had higher rMSSD than middle-aged individuals (Fig. 2E).

### rMSSD and ADI-RRB scores in ASD

**Fig. 3:**
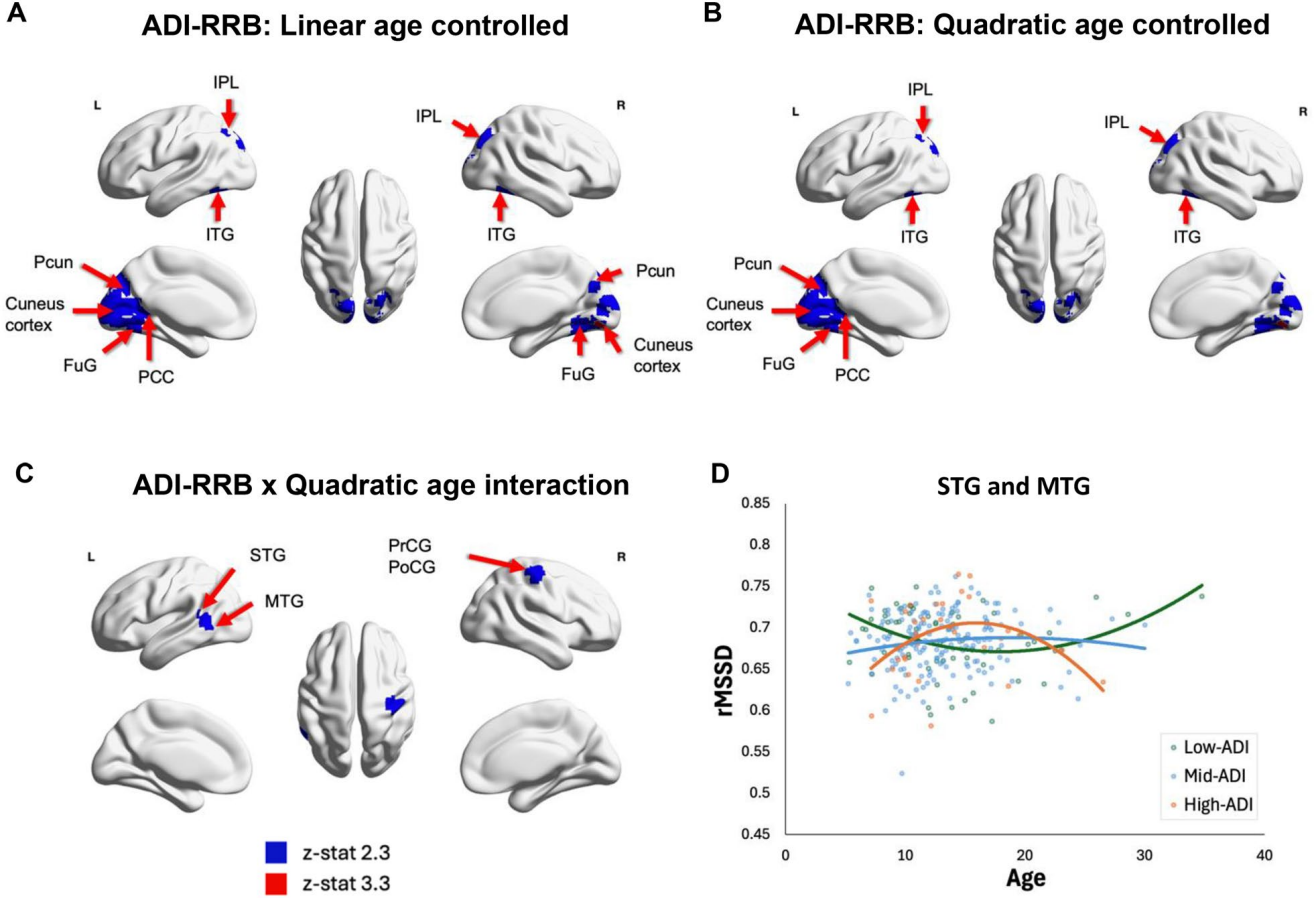
**A**, **B** Results from regression Model 5 and 6 showing brain areas showing a significant positive relationship between rMSSD and ADI-RRB scores while controlling linear (**A**) and quadratic age effects (**B**).** C** Results from regression Model 8 showing a significant quadratic Age x ADI-RRB interaction effect in the PrCG, PoCG, STG, and MTG (*p* < 0.05 cluster-wise corrected). **D** Specifically, individuals with high ADI-RRB scores showed an inverted U-shaped relationship between rMSSD and age while individuals with low ADI-RRB scores exhibit a U-shaped relationship between rMSSD and age. Individuals with mid-range ADI-RRB scores demonstrated a weak inverted-u shaped trend in the relationship between rMSSD and age. *ADI-RRB* repetitive restricted behavior indexed by Autism Diagnostic Interview, *rMSSD* root mean-square successive difference, *TD* typically developing, *ASD* autism spectrum disorder, *Pcun* precuneus, *FuG* fusiform gyrus, *PCC* posterior cingulate cortex, *IPL* inferior parietal lobule, *ITG* inferior temporal gyrus, *PoCG* postcentral gyrus, *PrCG* precentral gyrus, *STG* superior temporal gyrus, *MTG* middle temporal gyrus

When controlling for linear (Model 5) (Fig. 3A) and quadratic age (Model 6) (Fig. 3B) effects, we found significant positive relationship between rMSSD and ADI-RRB scores. This pattern was evident in clusters located in the PCC, inferior parietal lobule (IPL), precuneus (Pcun), inferior temporal gyrus (ITG), fusiform gyrus (FuG), and cuneus cortex at a voxel-wise uncorrected threshold of z > 2.3. A more stringent threshold (z > 3.3) additionally highlighted the FuG as a significant region.

While no significant age x RRB interaction was observed (Model 7), a quadratic age x RRB interaction (Model 8) was identified at a more lenient threshold (voxel-wise uncorrected, z > 2.3) in the precentral gyrus (PrCG), postcentral gyrus (PoCG), STG, and MTG region (Fig. 3C). Individuals with high ADI-RRB scores exhibited an inverted U-shaped rMSSD-age association, with low rMSSD levels observed in both younger and older individuals, and high rMSSD levels in middle-aged individuals. Those with mid-range ADI-RRB scores displayed a weak inverted U-shaped rMSSD-age association. In contrast, individuals with low ADI-RRB scores showed a U-shaped rMSSD-age association, with high rMSSD levels in younger and older individuals, but a low rMSSD levels in middle-aged individuals (Fig. 3D). These results show that relationships between rMSSD, age, and ADI-RRB scores may follow quadratic patterns in sensorimotor and temporal cortices. The scatterplots signifying the age x RRB interaction categorized the subjects into three groups based on their ADI-RRB scores (low, mid, high) using equal binning intervals for visualization purposes only (Fig. 3D).

### Reproducibility analysis

The reproducibility analysis conducted using the additional nuisance covariates of handedness, IQ, and gender yielded significant clusters of rMSSD values ASD < TD for Models 1 and 2, specifically in the TPJ, lingual gyrus, cuneus, STG, and MTG with an additional cluster was observed in the occipital cortex (Additional Fig. 1A and B). Model 3, run with the additional nuisance covariates, yielded significant differences between ASD and TD groups, with rMSSD values being higher in ASD compared to TD in areas such as the PCC, PHG, lingual gyrus, and occipital cortex, as observed in the original analysis. It also revealed additional significant clusters in the right Pcun (Additional Fig. 1C). Similarly, Model 4 run with additional covariates revealed significant group x quadratic age effects in clusters where rMSSD values were lower in ASD compared to TD (ASD < TD), specifically in the mPFC, consistent with the primary analysis. Additionally, significant clusters were observed in the PCC and orbitofrontal cortex (Additional Fig. 1D). This indicates that, after controlling for IQ, handedness, and gender, the categorical group differences and the age × group and quadratic age x group interactions remain largely consistent.

Models 5 and 6 run with the additional covariates yielded significant clusters indicating positive relationship between rMSSD and ADI-RRB scores in areas such as PCC, Pcun, IPL, FuG, ITG, and cuneus cortex as in primary analysis. Model 7 was not examined as there were no significant results in the main analysis. Finally, Model 8 run with additional covariates revealed significant effects indicating a quadratic relationship between rMSSD and the age × ADI-RRB interaction term, specifically in the left MTG, left ITG, right PoCG, and right PrCG, consistent with the primary analysis. However, the post hoc analysis also identified new significant clusters in the right MTG and right ITG. Overall, these results suggest that the relationship between rMSSD and ADI-RRB, after controlling for age and the quadratic age × ADI-RRB interaction, remains largely consistent even when accounting for IQ, handedness, and gender.

## Discussion

Brain signal variability is thought to be crucial for optimal cognitive and behavioral function [27] and is theorized to support the dynamic interplay between integrated and segregated brain states, which allows different brain regions to synchronize or operate independently depending on the specific task requirements [27]. Previous studies have focused on BOLD fMRI variability within the context of aging [6, 7, 28] and neurodevelopmental disorders [5, 13]. The current study investigated the age-related relationship between resting-state BOLD fMRI variability and repetitive behaviors in ASD in a large publicly available sample for the first time. The results show that rMSSD can differentiate between ASD and TD individuals using approaches that identify group differences with and without consideration of age. The results also show that restrictive and repetitive symptom severity in ASD as measured by the ADI-RRB scale is associated with rMSSD in both a linear and quadratic manner with age. Finally, controlling for demographics such as IQ, handedness, and gender in a subset of individuals did not affect the relationship between rMSSD and ADI-RRB scores. It also increased the spatial extent of categorical group differences, age x group, quadratic age x group effects, and the quadratic age x RRB interaction. Taken together, these results show how rMSSD may be used to identify differences in BSV between individuals with ASD and TD and how rMSSD may be used to identify associations with restrictive and repetitive behaviors in individuals with ASD.

### Group differences in brain signal variability

This pattern of BSV in TD individuals generally aligns with earlier investigations reporting a general linear decrease in BSV across the cortex in TD individuals across the lifespan [4]. This general decrease is thought to be associated with maturational processes that occur with the degradation of processing speed and cognitive abilities across old age [6, 9, 29]. However, caution should also be used when comparing the results of the current study against previous research, as the age ranges differ considerably. Still, it is unclear why individuals with ASD show the opposite pattern, where BSV increases across development. This divergence may be related to a number of factors such as differences in brain maturational processes in ASD compared with TD. For example, individuals with ASD show early developmental brain overgrowth compared with TD individuals [30]. Post-mortem examinations of brains from individuals with ASD show age-related decreases in Gamma-aminobutyric (GABA) acid associated with immune and inflammation responses [31]. Such findings have led to speculation that early neurobiological differences present in ASD from early age (e.g., 2 years old) results in a cascading effect where typical brain development is affected [32]. These early developmental deviations in individuals with ASD from TD individuals may contribute to the differences in BSV found in the current study.

Previously, Easson and McIntosh (2019) found no group differences between ASD and TD individuals and a positive relationship between MSSD and age across both ASD and TD individuals. In contrast, we found reduced BSV in individuals with ASD compared with TD individuals when controlling for age and quadratic age in temporal and occipital cortices including the TPJ, MTG, STG, cuneus cortex, and lingual gyrus. We also demonstrate an age × group interaction where there was a positive association between rMSSD and age in the lingual gyrus, PCC, PHG, and occipital cortex in individuals with ASD while the TD group showed a negative association between rMSSD and age. There was also a quadratic age × group interaction, where individuals with ASD exhibited a slight increase in rMSSD during childhood and older adulthood, followed by a significant drop in middle adulthood in the prefrontal cortex and paracingulate gyrus, whereas TD individuals exhibit higher rMSSD in childhood and older adulthood, and lower rMSSD for middle-aged individuals.

The divergence of our results from Easson and McIntosh (2019) could arise from a number of differences between the two studies such as the sample size, age range, and the use of a multivariate (e.g., Easson and McIntosh 2019) versus univariate approach (e.g., current study). Future research should attempt to clarify how such methodological differences may influence BSV when comparing ASD and TD, when exploring how age impacts such clinical differences, and how ASD symptom severity is related to BSV. These studies underscore the complex and dynamic nature of identifying age-related changes in brain variability related to the developmental trajectories of ASD and TD individuals.

### Restricted and repetitive behaviors and rMSSD

Easson and McIntosh (2019) previously identified a negative relationship between BSV and scores on the social responsiveness scale (SRS) across individuals with and without an ASD diagnosis. We investigated the relationship between rMSSD and repetitive behavior (ADI-RRB scores). While controlling for the main effects of linear and quadratic age, we found positive significant association between rMSSD and ADI-RRB scores. This pattern was evident in clusters located in the PCC, IPL, Pcun, ITG, FuG, and cuneus cortex which belong to DMN, salience and visual networks. While we found no significant linear age × ADI-RRB interaction effects, the quadratic age × ADI-RRB interaction revealed an inverted U-shaped relationship between rMSSD and age for individuals with higher repetitive behavior scores (i.e., lower rMSSD levels in both younger and older individuals, and higher rMSSD in middle-aged individuals). Those with mid-range repetitive behavior scores displayed a weak inverted U-shaped rMSSD-age association. In contrast, those with lower scores showed a U-shaped relationship between rMSSD and age (i.e., higher rMSSD levels in both younger and older individuals, and lower rMSSD in middle-aged individuals), particularly in the PrCG, PoCG, STG, and MTG region which belong to functional brain networks such as DMN, dorsal attention network (DAN), and sensorimotor network (SMN). These patterns highlight how the relationship between ASD symptom severity strongly depends on the clinical symptom, sample size, and methodological approach.

Our findings align with the neurobiological mechanisms underlying altered BSV and RRB in ASD. In ASD, an imbalance between excitation and inhibition—characterized by increased glutamate and decreased GABA—reduces the functional differentiation of brain processing systems, leading to excessive neural activity and increased noise. This heightened noise compromises the ability of the brain to process information accurately, reducing the reliability of neural representations [33, 34]. Studies in ASD rodent models have demonstrated that heightened excitatory and diminished inhibitory signaling can induce RRBs [35, 36]. Additionally, dopamine signaling plays a significant role in modulating BSV. Dopamine is integral to neuromodulation, regulating synaptic activity and modulating synaptic strength, influenced by factors such as the duration of dopamine receptor activation [37]. Depletion of dopamine in specific brain regions has been linked to increased BSV and reduced functional connectivity [38]. Altered dopamine signaling in ASD is linked to RRB-like symptoms, and studies report that dopamine agonist drugs can improve RRBs in individuals with ASD [39]. Overall, these neurobiological aspects may explain the relationship between the increased BSV and severity of RRBs in the ASD group.

The interplay between age, BSV, and RRB in ASD aligns with prior neuroimaging studies, which have reported associations between RRB and atypical functional connectivity in multiple resting-state networks across different age groups in ASD [40–43]. Abbott and colleagues reported an imbalance of cortico-striatal intrinsic functional connectivity in children and adolescents (8–17 years) with ASD. This imbalance was characterized by increased functional connectivity in limbic circuits and reduced functional connectivity in frontoparietal and motor circuits and was associated with RRB [41]. Weng and colleagues reported hypo-connectivity between PCC, mPFC, temporal lobes, and superior frontal gyrus (SFG) associated with RRB in adolescence [43]. Studies in adults with ASD revealed hyperconnectivity between PCC and the right parahippocampal gyrus [42]. Overall, these studies demonstrate that links between functional connectivity and the RRB behavioral phenotype changes from childhood to adulthood in ASD. These findings, coupled with the results of our current analysis, imply that the brain regions from DMN, DAN, SMN, and salience network exhibit age-related atypical BSV, and functional connectivity trajectories associated with RRBs in ASD. Previous studies investigating the relationship between functional connectivity and BSV have yielded mixed findings. Some report reduced functional connectivity in brain regions with increased BSV [38], while others find a positive correlation between BSV and functional connectivity [44]. Further research is required to understand the age-related dynamics of BOLD variability and its relationship with functional connectivity, especially in ASD.

The current results support the notion that there is a relationship between heightened BSV and the occurrence of RRBs; however, the direction of causality remains uncertain. It is unclear whether increased BSV precedes and influences the development of RRBs or if the observed variability patterns are a consequence of the RRBs themselves (e.g., potentially arising from ongoing efforts to manage and control them). RRBs have been observed to be less prevalent and less severe in older age groups compared with younger individuals [45, 46]. It is plausible that the increase in BSV with age among individuals with higher RRBs may serve a compensatory purpose in adulthood. These changes may compensate for age-related reductions in network complexity and integration or might indicate dysfunctional signal variability. For example, stochastic resonance (SR) may play a role in the observed increase in BSV with age, potentially reflecting a compensatory mechanism where noise is optimized to maintain neural function and processing efficiency despite age-related declines. This increase in BSV could also indicate a shift in SR dynamics, where greater external or internal noise becomes necessary to support the same level of neural integration and signal detection in older individuals, particularly those with higher levels of RRBs [47–49].

In contrast to individuals with elevated RRBs, we observed a reduction in rMSSD associated with lower RRB symptoms from childhood to adulthood. These findings suggest that these individuals have potentially maintained neural efficiency over time. The brains of individuals with lower RRB scores might be more capable of maintaining stable and efficient neural networks as they age, avoiding unnecessary increases in BSV that could potentially disrupt cognitive function. Future work should elucidate the relationships between RRBs and the maturation of functional brain networks across development using longitudinal datasets to enhance early risk assessment, inform developmental models of ASD pathogenesis, and provide a neurophysiological foundation for novel interventions focused on RRBs.

### Limitations

There are a few limitations important to note in the present study. First, we employed a cross-sectional approach to examine age-related associations between variability and repetitive behaviors, which limits our ability to infer developmental trajectories [50]. Additionally, despite the demographic matching of groups, a cross-sectional design cannot disentangle the effects of a condition from its underlying causes. An important consideration of our cross-sectional study is the potential for misinterpreting age-related correlations, as it assumes homogeneity among younger and older individuals with the same diagnosis, disregarding the distinct characteristics associated with developmental psychopathologies that vary with age. Moreover, the inherent constraint of cross-sectional studies lies in their inability to capture individual longitudinal changes, impeding our comprehension of how the illness and related imaging measures evolve. Future research employing longitudinal designs could provide valuable insights into how the relationship between BSV and repetitive behaviors in individuals with ASD evolves.

Although our study includes participants aged 5 to 50, the number of individuals above 30 is limited due to constraints in the ABIDE dataset. Specifically, our primary analysis involved only 22 participants over 30, and the oldest participant did not exceed 30 for the ADI-RRB analysis. This skew towards childhood, adolescence, and young adulthood in our study provides an opportunity for a more detailed examination of these developmental stages. However, the limited number of older participants suggests that age x diagnosis interactions might be influenced predominantly by the few older individuals included, potentially affecting the generalizability of our findings across the entire age range. Future research should consider employing datasets with a broader and more evenly distributed age range. This would ensure a more comprehensive analysis and provide better insights into how the relationships between BSV, age, and ASD symptomatology evolve across the lifespan.

Another limitation in our study concerns potential heterogeneity within the ASD group [51]. We primarily focused on right-handed, high-functioning adult males, which could not account for all between-participant differences. Recent studies have suggested that genetic patterns may influence neural responses in autism [52, 53], and there is substantial heterogeneity among individuals with ASD [54]. To gain a more comprehensive understanding, future investigations should explore these observations in more genetically and behaviorally homogeneous subgroups of ASD individuals.

While we used the ADI-RRB scale to explore the age-related relationship between BSV and repetitive behavior in ASD, the limited availability of additional RRB measures and the smaller number of participants with these measures in the ABIDE dataset may result in an incomplete assessment of RRBs. Consequently, our findings may not capture all the nuanced relationships between BSV and RRB. Future research should consider employing a broader array of RRB assessments with a larger sample size to enhance the power and robustness of the findings. DSM-5 [55] classifies RRBs into four subtypes: (a) stereotyped or repetitive motor movements, (b) inflexible adherence to routines, (c) highly restricted, fixated interests that are abnormal in intensity or focus, and (d) hyper- or hypo-reactivity to sensory input or unusual interests in sensory aspects of the environment. Given the heterogeneity of these repetitive behaviors, there exist considerable challenges in achieving a thorough understanding and comprehensive investigation of this phenotype. Future research should aim to address these gaps and explore the associations between BSV and specific subcategories of repetitive behaviors in individuals with ASD.

Lastly, in our study, rMSSD values ranged from 0.3 to 1. The range of MSSD values depends strongly on the TR, as longer TRs allow for more changes of the BOLD signal between images. Thus, rMSSD is not capped at a maximum value of 1, but it is dependent on the TR of the fMRI scan. We previously showed that the range of MSSD for a fast TR (0.645 s) is different than the range of MSSD for a slow TR (1.4 s); additionally, the strength of linear and quadratic effects may depend on the TR [56]. Thus, the choice of TR could have influenced the linear and quadratic effects observed in our analysis. Further studies should evaluate the influence of different TR values on age-related BSV in ASD to optimize fMRI acquisition and improve assessments of brain signal variability in ASD.

## Conclusions

The current study reveals categorical and dimensional relationships between BSV, age, and RRB severity across ASD and TD participants. When exploring categorical group differences in BSV, we found that variability in the TPJ, temporal cortex, and occipital cortex is significantly lower in ASD compared with TD. For the age x group interaction, we observed a linear increase in variability in DMN and visual network nodes with age in ASD with a linear decrease in variability with age in TD individuals. In contrast, the quadratic age × group interaction showed an inverted U-shaped rMSSD-age relationship in ASD and a U-shaped rMSSD-age relationship in TD in the DMN and nodes. We observed a continuum of relationships when examining variability concerning predictor variables like repetitive behavioral severity. We found a significant positive relationship between rMSSD and ADI-RRB, controlling for linear and quadratic age, within nodes of the DMN, salience, and visual networks. Although the linear age x ADI-RRB interaction showed no significant findings, the quadratic age x ADI-RRB interaction revealed an inverted U-shaped relationship for individuals with higher repetitive behavior scores. Those with mid-range repetitive behavior scores displayed a weak inverted U-shaped rMSSD-age association. Whereas those with lower scores demonstrated U-shaped relationship, particularly in the brain regions of DMN, DAN, and SMN. These findings highlight distinctive age-related BSV patterns linked to repetitive behaviors in ASD, contributing to the growing literature on atypical neural variability in ASD.

## Supplementary Information

**Additional file 1**: Reproducibility analysis. Additional figures 1 and 2 demonstrate that the voxel wise effects in all the analysis remained consistent even after accounting covariates such as IQ, gender, and handedness.

**Additional file 2**: Data availability. This file contains the subject IDs obtained from the ABIDE dataset, which were utilized for our analysis.

## Data Availability

The datasets supporting the conclusions of this article are available in the Autism Brain Imaging Data Exchange (ABIDE, http://fcon_1000.projects.nitrc.org/indi/abide/). Additionally, the subject IDs referenced in the article are provided in the supplementary Additional file 2.

## Abbreviations

BSV: Brain signal variability
ASD: Autism spectrum disorder
TD: Typically developing
RRB: Repetitive and restricted behavior
ABIDE: Autism brain imaging data exchange
ADI-R: Autism diagnostic interview-revised
rMSSD: Root mean square successive difference
PoCG: Postcentral gyrus
PrCG: Precentral gyrus
Pcun: Precuneus
PHG: Parahippocampal gyrus
STG: Superior temporal gyrus
ITG: Inferior temporal gyrus
MFG: Middle frontal gyrus
PCC: Posterior cingulate cortex
MTG: Middle temporal gyrus
SFG: Superior frontal gyrus
FuG: Fusiform gyrus
mFC: Medial frontal cortex
IPL: Inferior parietal lobule
IFG: Inferior parietal gyrus
BOLD: Blood-oxygen level-dependent
fMRI: Functional magnetic resonance imaging
TR: Repetition time
IQ: Intelligence quotient
FD: Framewise displacement
ADOS-2: Autism diagnostic observation schedule 2
DSM-IV: Diagnostic and Statistical Manual of Mental Disorders (4th edition)
DPARSF: Data Processing Assistant for Resting-State fMRI toolbox
ICA: Independent component analysis
FSL: FMRIB Software Library
OLS: Ordinary least squares
DV: Dependent variable
GRF: Gaussian Random Field
DMN: Default mode network
CEN: Central executive network
DAN: Dorsal attention network
SMN: Sensorimotor network
TPJ: Temporal-parietal junction

## References

[1] Uddin LQ. Bring the noise: reconceptualizing spontaneous neural activity. Berlin: Elsevier; 2020. 10.1016/j.tics.2020.06.003. 10.1016/j.tics.2020.06.003 PMC7429348 32600967

[2] Beck JM, et al. Probabilistic population codes for Bayesian decision making. Neuron. 2008;60(6):1142–52. 10.1016/J.NEURON.2008.09.021. 19109917 10.1016/j.neuron.2008.09.021 PMC2742921

[3] Garrett DD, Epp SM, Perry A, Lindenberger U. Local temporal variability reflects functional integration in the human brain. Neuroimage. 2018;183:776–87. 10.1016/J.NEUROIMAGE.2018.08.019. 30149140 10.1016/j.neuroimage.2018.08.019

[4] Nomi JS, Bolt TS, Chiemeka Ezie CE, Uddin LQ, Heller AS. Moment-to-moment BOLD signal variability reflects regional changes in neural flexibility across the lifespan. J Neurosci. 2017;37(22):5539–48. 10.1523/JNEUROSCI.3408-16.2017. 28473644 10.1523/JNEUROSCI.3408-16.2017 PMC5452342

[5] Easson AK, McIntosh AR. BOLD signal variability and complexity in children and adolescents with and without autism spectrum disorder. Dev Cogn Neurosci. 2019;36: 100630. 10.1016/J.DCN.2019.100630. 30878549 10.1016/j.dcn.2019.100630 PMC6969202

[6] Garrett DD, Kovacevic N, McIntosh AR, Grady CL. Blood oxygen level-dependent signal variability is more than just noise. J Neurosci. 2010;30(14):4914–21. 10.1523/JNEUROSCI.5166-09.2010. 20371811 10.1523/JNEUROSCI.5166-09.2010 PMC6632804

[7] Garrett DD, Kovacevic N, McIntosh AR, Grady CL. The modulation of BOLD variability between cognitive states varies by age and processing speed. Cereb Cortex. 2013;23(3):684–93. 10.1093/CERCOR/BHS055. 22419679 10.1093/cercor/bhs055 PMC3823571

[8] Garrett DD, McIntosh AR, Grady CL. Brain signal variability is parametrically modifiable. Cereb Cortex. 2014;24(11):2931–40. 10.1093/CERCOR/BHT150. 23749875 10.1093/cercor/bht150 PMC4193462

[9] Garrett DD, et al. Amphetamine modulates brain signal variability and working memory in younger and older adults. Proc Natl Acad Sci USA. 2015;112(24):7593–8. 10.1073/pnas.1504090112. 26034283 10.1073/pnas.1504090112 PMC4475975

[10] Grady CL, Garrett DD. Brain signal variability is modulated as a function of internal and external demand in younger and older adults. Neuroimage. 2018;169:510–23. 10.1016/j.neuroimage.2017.12.031. 29253658 10.1016/j.neuroimage.2017.12.031

[11] Samanez-Larkin GR, Kuhnen CM, Yoo DJ, Knutson B. Variability in nucleus accumbens activity mediates age-related suboptimal financial risk taking. J Neurosci. 2010;30(4):1426–34. 10.1523/JNEUROSCI.4902-09.2010. 20107069 10.1523/JNEUROSCI.4902-09.2010 PMC2821055

[12] Goodman ZT, et al. Brain signal variability and executive functions across the life span. Netw Neurosci. 2024;8(1):226–40. 10.1162/NETN_A_00347. 38562287 10.1162/netn_a_00347 PMC10918754

[13] Nomi JS, Schettini E, Voorhies W, Bolt TS, Heller AS, Uddin LQ. Resting-state brain signal variability in prefrontal cortex is associated with ADHD symptom severity in children. Front Hum Neurosci. 2018. 10.3389/FNHUM.2018.00090. 29593515 10.3389/fnhum.2018.00090 PMC5857584

[14] Månsson KNT, Waschke L, Manzouri A, Furmark T, Fischer H, Garrett DD. Moment-to-moment brain signal variability reliably predicts psychiatric treatment outcome. Biol Psychiatry. 2022;91(7):658–66. 10.1016/j.biopsych.2021.09.026. 34961621 10.1016/j.biopsych.2021.09.026

[15] Li L, et al. Altered brain signal variability in patients with generalized anxiety disorder. Front Psychiatry. 2019;10:84. 10.3389/FPSYT.2019.00084/BIBTEX. 30886589 10.3389/fpsyt.2019.00084 PMC6409298

[16] Di Martino A, et al. Enhancing studies of the connectome in autism using the autism brain imaging data exchange II. Sci Data. 2017. 10.1038/SDATA.2017.10. 28291247 10.1038/sdata.2017.10 PMC5349246

[17] Di Martino A, et al. The autism brain imaging data exchange: towards a large-scale evaluation of the intrinsic brain architecture in autism. Mol Psychiatry. 2014;19(6):659–67. 10.1038/MP.2013.78. 23774715 10.1038/mp.2013.78 PMC4162310

[18] Power JD, Barnes KA, Snyder AZ, Schlaggar BL, Petersen SE. Spurious but systematic correlations in functional connectivity MRI networks arise from subject motion. Neuroimage. 2012;59(3):2142–54. 10.1016/j.neuroimage.2011.10.018. 22019881 10.1016/j.neuroimage.2011.10.018 PMC3254728

[19] Chao-Gan Y, Yu-Feng Z. DPARSF: a MATLAB toolbox for ‘pipeline’ data analysis of resting-state fMRI. Front Syst Neurosci. 2010. 10.3389/FNSYS.2010.00013. 20577591 10.3389/fnsys.2010.00013 PMC2889691

[20] Salimi-Khorshidi G, Douaud G, Beckmann CF, Glasser MF, Griffanti L, Smith SM. Automatic denoising of functional MRI data: combining independent component analysis and hierarchical fusion of classifiers. Neuroimage. 2014;90:449–68. 10.1016/J.NEUROIMAGE.2013.11.046. 24389422 10.1016/j.neuroimage.2013.11.046 PMC4019210

[21] Griffanti L, et al. ICA-based artefact removal and accelerated fMRI acquisition for improved resting state network imaging. Neuroimage. 2014;95:232–47. 10.1016/J.NEUROIMAGE.2014.03.034. 24657355 10.1016/j.neuroimage.2014.03.034 PMC4154346

[22] Feis RA, et al. ICA-based artifact removal diminishes scan site differences in multi-center resting-state fMRI. Front Neurosci. 2015;9:153425. 10.3389/FNINS.2015.00395/BIBTEX. 10.3389/fnins.2015.00395 PMC4621866 26578859

[23] Fortin JP, et al. Harmonization of cortical thickness measurements across scanners and sites. Neuroimage. 2018;167:104. 10.1016/J.NEUROIMAGE.2017.11.024. 29155184 10.1016/j.neuroimage.2017.11.024 PMC5845848

[24] von Neumann J. Distribution of the ratio of the mean square successive difference to the variance. Ann Math Stat. 1941;12(4):367–95. 10.1214/AOMS/1177731677.

[25] Woo CW, Krishnan A, Wager TD. Cluster-extent based thresholding in fMRI analyses: pitfalls and recommendations. Neuroimage. 2014;91:412. 10.1016/J.NEUROIMAGE.2013.12.058. 24412399 10.1016/j.neuroimage.2013.12.058 PMC4214144

[26] Eklund A, Nichols TE, Knutsson H. Cluster failure: why fMRI inferences for spatial extent have inflated false-positive rates. Proc Natl Acad Sci USA. 2016;113(28):7900–5. 10.1073/PNAS.1602413113. 27357684 10.1073/pnas.1602413113 PMC4948312

[27] Garrett DD, Samanez-Larkin GR, MacDonald SWS, Lindenberger U, McIntosh AR, Grady CL. Moment-to-moment brain signal variability: a next frontier in human brain mapping? Neurosci Biobehav Rev. 2013;37(4):610–24. 10.1016/J.NEUBIOREV.2013.02.015. 23458776 10.1016/j.neubiorev.2013.02.015 PMC3732213

[28] Tognoli E, Kelso JAS. The metastable brain. Neuron. 2014;81(1):35–48. 10.1016/J.NEURON.2013.12.022. 24411730 10.1016/j.neuron.2013.12.022 PMC3997258

[29] Garrett DD, Kovacevic N, McIntosh AR, Grady CL. The importance of being variable. J Neurosci. 2011;31(12):4496–503. 10.1523/JNEUROSCI.5641-10.2011. 21430150 10.1523/JNEUROSCI.5641-10.2011 PMC3104038

[30] Garrett DD, Kovacevic N, McIntosh AR, Grady CL. The modulation of BOLD variability between cognitive states varies by age and processing speed. Cereb Cortex. 2013;23(3):684–93. 22419679 10.1093/cercor/bhs055 PMC3823571

[31] Girault JB, Piven J. The neurodevelopment of autism from infancy through toddlerhood. Neuroimaging Clin N Am. 2020;30(1):97. 10.1016/J.NIC.2019.09.009. 31759576 10.1016/j.nic.2019.09.009 PMC6878903

[32] Zhang P, Omanska A, Ander BP, Gandal MJ, Stamova B, Schumann CM. Neuron-specific transcriptomic signatures indicate neuroinflammation and altered neuronal activity in ASD temporal cortex. Proc Natl Acad Sci USA. 2023;120(10): e2206758120. 10.1073/PNAS.2206758120/SUPPL_FILE/PNAS.2206758120.SD14.XLSX. 36862688 10.1073/pnas.2206758120 PMC10013873

[33] Courchesne E, Pierce K. Why the frontal cortex in autism might be talking only to itself: local over-connectivity but long-distance disconnection. Curr Opin Neurobiol. 2005;15(2):225–30. 10.1016/J.CONB.2005.03.001. 15831407 10.1016/j.conb.2005.03.001

[34] Rubenstein JLR, Merzenich MM. Model of autism: increased ratio of excitation/inhibition in key neural systems. Genes Brain Behav. 2003;2(5):255. 10.1034/J.1601-183X.2003.00037.X. 14606691 10.1034/j.1601-183x.2003.00037.x PMC6748642

[35] Nelson SB, Valakh V. Excitatory/inhibitory balance and circuit homeostasis in autism spectrum disorders. Neuron. 2015;87(4):684. 10.1016/J.NEURON.2015.07.033. 26291155 10.1016/j.neuron.2015.07.033 PMC4567857

[36] Rinaldi T, Kulangara K, Antoniello K, Markram H. Elevated NMDA receptor levels and enhanced postsynaptic long-term potentiation induced by prenatal exposure to valproic acid. Proc Natl Acad Sci. 2007. 10.1073/pnas.0704391104. 17675408 10.1073/pnas.0704391104 PMC1948920

[37] Gogolla N, LeBlanc JJ, Quast KB, Südhof TC, Fagiolini M, Hensch TK. Common circuit defect of excitatory-inhibitory balance in mouse models of autism. J Neurodev Disord. 2009;1(2):172–81. 10.1007/S11689-009-9023-X. 20664807 10.1007/s11689-009-9023-x PMC2906812

[38] Seamans JK, Yang CR. The principal features and mechanisms of dopamine modulation in the prefrontal cortex. Prog Neurobiol. 2004;74(1):1–58. 15381316 10.1016/j.pneurobio.2004.05.006

[39] Shafiei G, et al. Dopamine signaling modulates the stability and integration of intrinsic brain networks. Cereb Cortex (New York, NY). 2019;29(1):397. 10.1093/CERCOR/BHY264. 10.1093/cercor/bhy264 PMC6294404 30357316

[40] Mandic-Maravic V, Grujicic R, Milutinovic L, Munjiza-Jovanovic A, Pejovic-Milovancevic M. Dopamine in autism spectrum disorders—focus on D2/D3 partial agonists and their possible use in treatment. Front Psychiatry. 2021;12: 787097. 10.3389/FPSYT.2021.787097. 35185637 10.3389/fpsyt.2021.787097 PMC8850940

[41] McKinnon CJ, et al. Restricted and repetitive behavior and brain functional connectivity in infants at risk for developing autism spectrum disorder. Biol Psychiatry Cogn Neurosci Neuroimaging. 2019;4(1):50–61. 10.1016/J.BPSC.2018.09.008. 30446435 10.1016/j.bpsc.2018.09.008 PMC6557405

[42] Abbott A, et al. Repetitive behaviors in autism are linked to imbalance of corticostriatal connectivity: a functional connectivity MRI study. Soc Cogn Affect Neurosci. 2018;13(1):32–42. 10.1093/scan/nsx129. 29177509 10.1093/scan/nsx129 PMC5793718

[43] Monk CS, et al. Abnormalities of intrinsic functional connectivity in autism spectrum disorders. Neuroimage. 2009;47(2):764–72. 10.1016/J.NEUROIMAGE.2009.04.069. 19409498 10.1016/j.neuroimage.2009.04.069 PMC2731579

[44] Weng SJ, et al. Alterations of resting state functional connectivity in the default network in adolescents with autism spectrum disorders. Brain Res. 2010;1313:202–14. 10.1016/J.BRAINRES.2009.11.057. 20004180 10.1016/j.brainres.2009.11.057 PMC2818723

[45] Baracchini G, et al. “The biological role of local and global fMRI BOLD signal variability in human brain organization,” bioRxiv, Oct. 2023, 10.1101/2023.10.22.563476. 10.1038/s41467-026-68700-0 PMC12960827 41618090

[46] Seltzer MM, Krauss MW, Shattuck PT, Orsmond G, Swe A, Lord C. The symptoms of autism spectrum disorders in adolescence and adulthood. J Autism Dev Disord. 2003;33(6):565–81. 10.1023/B:JADD.0000005995.02453.0B. 14714927 10.1023/b:jadd.0000005995.02453.0b

[47] Esbensen AJ, Seltzer MM, Lam KSL, Bodfish JW. Age-related differences in restricted repetitive behaviors in autism spectrum disorders. J Autism Dev Disord. 2009;39(1):57–66. 10.1007/S10803-008-0599-X. 18566881 10.1007/s10803-008-0599-x PMC2605515

[48] Li S-C, von Oertzen T, Lindenberger U. A neurocomputational model of stochastic resonance and aging. Neurocomputing. 2006;69(13–15):1153–560. 10.1016/j.neucom.2005.06.015.

[49] Lugo E, Doti R, Faubert J. Ubiquitous crossmodal stochastic resonance in humans: auditory noise facilitates tactile, visual and proprioceptive sensations. PLoS ONE. 2008. 10.1371/JOURNAL.PONE.0002860. 18682745 10.1371/journal.pone.0002860 PMC2481403

[50] McDonnell MD, Abbott D. What is stochastic resonance? Definitions, misconceptions, debates, and its relevance to biology. PLoS Comput Biol. 2009. 10.1371/JOURNAL.PCBI.1000348. 19562010 10.1371/journal.pcbi.1000348 PMC2660436

[51] Kraemer HC, Yesavage JA, Taylor JL, Kupfer D. How can we learn about developmental processes from cross-sectional studies, or can we? Am J Psychiatry. 2000;157(2):163–71. 10.1176/APPI.AJP.157.2.163. 10671382 10.1176/appi.ajp.157.2.163

[52] Jack A, Pelphrey KA. Annual research review: understudied populations within the autism spectrum—current trends and future directions in neuroimaging research. J Child Psychol Psychiatry. 2017;58(4):411–35. 10.1111/JCPP.12687. 28102566 10.1111/jcpp.12687 PMC5367938

[53] Watanabe T, et al. Oxytocin receptor gene variations predict neural and behavioral response to oxytocin in autism. Soc Cogn Affect Neurosci. 2017;12(3):496–506. 10.1093/scan/nsw150. 27798253 10.1093/scan/nsw150 PMC5390696

[54] Rudie JD, et al. Autism-associated promoter variant in MET impacts functional and structural brain networks. Neuron. 2012;75:904–15. 10.1016/j.neuron.2012.07.010. 22958829 10.1016/j.neuron.2012.07.010 PMC3454529

[55] Tian J, Gao X, Yang L. Repetitive restricted behaviors in autism spectrum disorder: from mechanism to development of therapeutics. Front Neurosci. 2022. 10.3389/FNINS.2022.780407. 35310097 10.3389/fnins.2022.780407 PMC8924045

[56] American Psychiatric Association. Diagnostic and statistical manual of mental disorders. 5th ed. Arlington: American Psychiatric Association; 2013.

