# Supplementary material for: Age-related changes in brain signal variability in autism spectrum disorder
Source: Mol Autism. 2025 Feb 8;16:8. doi: 10.1186/s13229-024-00631-3 (PMC11806755; doi:10.1186/s13229-024-00631-3)
Supplement: Supplementary file 1 — Additional file 1: Reproducibility analysis. Additional figures 1 and 2 demonstrate that the voxel wise effects in all the analysis remained consistent even after accounting covariates such as IQ, gender, and handedness. [file 13229_2024_631_MOESM1_ESM.docx]

**Additional file 1**

**Table 1**

|  | Site | ASD (n) | TD  (n) | Gender  Male (female) | | IQ  Range (mean ± SD) | | Handedness  n (Right/Left/Ambi.) | |
| --- | --- | --- | --- | --- | --- | --- | --- | --- | --- |
|  |  |  |  | ASD | TD | ASD | TD | ASD | TD |
| ABIDE  I | NYU | 62 | 83 | 54 (8) | 62 (21) | 84 – 148  (109 ±15) | 80 - 129  (110± 11) | 6 (R) 13 (L) 43 (A) | na (R) 16 (L) 67 (A) |
|  | SDSU | 12 | 22 | 12 (na) | 16 (6) | 81 – 141  (111 ± 13) | 88 - 126  (106 ± 11) | 11 (R) 1 (L) na (A) | 19 (R) 3 (L) na (A) |
|  | TCD | 23 | 24 | 23 (na) | 24 (na) | 72 - 135  (109± 15) | 89 - 133  (110± 12) | 23 (R) na (L) na (A) | 24 (R) 0 (L) na (A) |
|  | UM | 46 | 64 | 38 (8) | 50 (14) | 77 – 147.5 (107± 14) | 85 - 129  (109 ± 11) | 38 (R) 7 (L) 1 (A) | 57 (R) 7 (L) na (A) |
|  | USM | 41 | 35 | 41 (na) | 35 (na) | 80 – 132  (105± 14) | 89 - 128  (111 ± 11) | 4 (R) 3 (L) 34 (A) | 2 (R) 2 (L) 31 (A) |
|  | YALE | 25 | 27 | 19 (6) | 19 (8) | 41 - 141  (95± 21) | 73 - 137  (104 ± 16) | 20 (R) 5 (L) na (A) | 23 (R) 4 (L) na (A) |
| ABIDE  II | ETH | 8 | 23 | 8 (na) | 23 (na) | 82 - 123  (109 ± 14) | 100 - 133 (117± 10) | 8 (R) na (L) na (A) | 23 (R) na (L) na (A) |
|  | GU | 41 | 50 | 36 (5) | 24 (26) | 92 - 149  (119 ± 15) | 91 - 149  (121± 14) | 33 (R) 8 (L) 0 (A) | 47 (R) 3 (L) na (A) |
|  | NYU | 25 | 18 | 22 (3) | 17 (1) | 74 - 138  (106± 19) | 91 - 128  (112± 12) | 19 (R) 2 (L) 4 (A) | 18 (R) 0 (L) na (A) |
|  | SDSU | 32 | 24 | 25 (7) | 22 (2) | 66 - 130  (100± 15) | 79 - 125  (103± 12) | 26 (R) 4 (L) 2 (A) | 20 (R) 1 (L) 3 (A) |
|  | UCD | 15 | 14 | 13 (2) | 10 (4) | 92 - 130  (105 ± 11) | 92 - 128  (113 ± 11) | 14 (R) na (L) 1 (A) | 14 (R) na (L) na (A) |
|  | USM | 6 | 5 | 6 (na) | 4 (1) | 87 - 134  (109 ± 19) | 99 - 118  (109 ± 8) | 6 (R) na (L) na (A) | 5 (R) na (L) na (A) |
| Total | | 336 | 389 |  |  |  |  |  |  |

*SD: Standard deviation, Ambi: Ambidexters, na = not applicable*

**Analyses controlling for covariates: gender, handedness, and IQ**

To evaluate the potential influence of IQ, handedness, and gender on the voxel-wise effects related to age-related group differences in rMSSD and the relationship between rMSSD and repetitive behavior, we performed ﻿eight post hoc regression analyses using the FSL GLM model.

The first and second post hoc regression models examined group differences in BSV while controlling for used linear age (mean-centered) (**Additional Figure 1A**), and quadratic age effects (squared mean-centered age) (**Additional Figure 1B**) with FD, handedness, IQ, and gender as nuisance covariates. These analyses yielded significant clusters of rMSSD values ASD < TD, specifically in the temporo-parietal junction (TPJ), lingual gyrus, cuneus, precuneus cortex (Pcun), superior temporal gyrus (STG), and medial temporal gyrus (MTG) with an additional cluster was observed in the occipital cortex. Notably, the majority of these clusters overlap with those identified in the primary analysis, which was conducted without these covariates. This demonstrates that significant voxel-wise effects persisted after accounting for IQ, handedness, or gender.

The third *post-hoc* regression model assessed the robustness of voxel-wise effects observed for the analysis exploring influence of the linear-age x group interaction on BSV group differences, accounting for IQ, gender, and handedness. This model included the linear-age x group interaction as the regressor of interest, with linear age, FD, handedness, IQ, and gender as additional nuisance covariates. This analysis yielded significant clusters of rMSSD values ASD > TD. While the directionality of the effects is conserved across both the post-hoc and primary analyses, the primary analysis revealed significant clusters in the posterior cingulate cortex (PCC), para-hippocampal gyrus (PHG), lingual gyrus and occipital cortex. The post-hoc analysis identified additional significant clusters in the right Pcun (**Additional Figure 1C**). This demonstrates that significant voxel-wise effects persisted after accounting for IQ, handedness, or gender.

The fourth post-hoc regression model evaluated the robustness of voxel-wise effects for the quadratic-age x group interaction, also considering IQ, gender, and handedness. This model used the quadratic-age x group interaction as the regressor of interest, with linear age, quadratic age, linear age x group interaction term, FD, handedness, IQ, and gender as nuisance covariates. The *post hoc* analysis revealed significant clusters where rMSSD values were lower in ASD than TD (ASD < TD), specifically in the medial prefrontal frontal cortex (mPFC), consistent with the primary analysis, along with additional significant clusters in the PCC and orbitofrontal cortex. (**Additional Figure 1D**). This demonstrates that the significant voxel-wise effects for the quadratic age x group interaction remained robust even after accounting for IQ, handedness, and gender.

The fifth and sixth post-hoc regression models examined whether the voxel-wise effects between rMSSD and ADI-RRB scores remained significant when adjusting for IQ, gender, and handedness. The fifth model controlled for linear age, while the sixth model controlled for both linear and quadratic age. In both models, ADI-RRB scores were the regressor of interest, with FD, handedness, IQ, and gender as nuisance covariates. These analyses, similar to the primary analysis, yielded significant clusters showing a positive relationship between rMSSD and ADI-RRB scores in the PCC, Pcun, inferior parietal lobule (IPL), inferior temporal gyrus (ITG), fusiform gyrus (FuG), and cuneus cortex. This demonstrates that significant voxel-wise effects persisted after accounting for IQ, handedness, or gender.

The seventh post hoc regression model investigated whether the voxel-wise effects pertaining to the influence of interaction between quadratic age and ADI-RRB on rMSSD remained statistically significant when adjusting for the potential confounding variables of IQ, gender, and handedness. This model included the quadratic age by ADI-RRB interaction term as the regressor of interest, while controlling for quadratic age and the linear age by ADI-RRB interaction term as additional covariates. FD, handedness, IQ, and gender were also included as nuisance covariates. This analysis revealed significant clusters showing a negative relationship between rMSSD and the quadratic age × ADI-RRB interaction term, particularly in the left MTG, left ITG, right postcentral gyrus (PoCG), and right precentral gyrus (PrCG), consistent with the primary analysis. Additionally, the post hoc analysis identified new significant clusters in the right MTG and right ITG. Overall, these results indicate that, while the linear age x ADI-RRB interaction effect on rMSSD is not robust, the quadratic age x ADI-RRB interaction effect remains significant after accounting for these additional factors.

**A B**

**BSV group difference:**

**Quadratic age controlled**

**BSV group difference:**

**Linear age controlled**


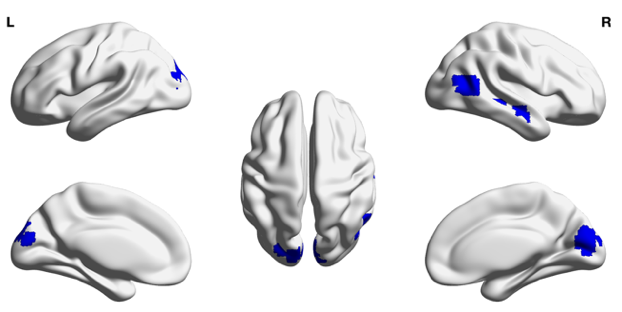

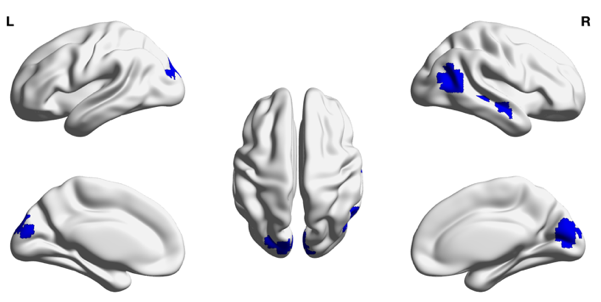


**Group x Quadratic age interaction**

**C D**

**Group x Linear age interaction**


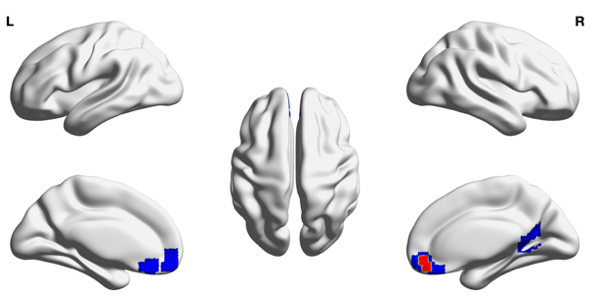

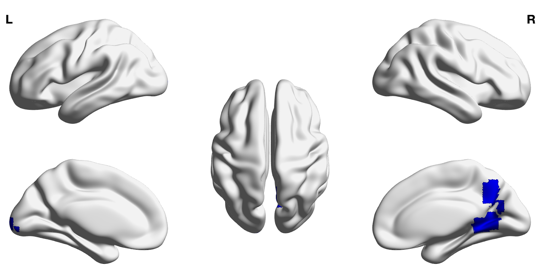


*
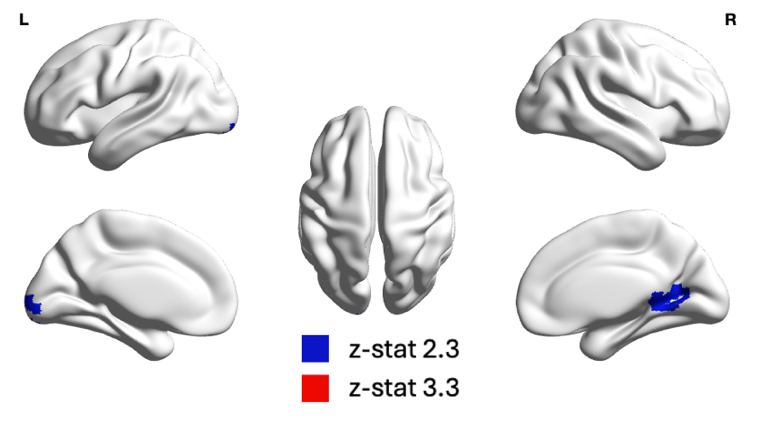
*

**Figure 1 | BSV group difference**

To examine the impact of IQ, gender, and handedness on BSV group differences, we performed post-hoc regression analysis using the FSL GLM model. We conducted this analysis using a sample of 336 participants with ASD and 389 TD participants, as the remaining subjects lacked information on either gender or handedness. Linear (**A**) and Quadratic Age main effect (**B**)| Significant clusters of rMSSD values ASD < TD (voxel-wise uncorrected, (z > 2.3 & 3.3) and cluster-wise corrected (p < 0.05). Linear Age x group interaction effect (**C**)| Significant clusters of rMSSD values ASD > TD (voxel-wise uncorrected, (z > 2.3 & 3.3) and cluster-wise corrected (p < 0.05)). Quadratic age x group interaction effect (**D**)| Significant clusters of rMSSD values ASD < TD in (voxel-wise uncorrected, (z > 2.3) and cluster-wise corrected (p < 0.05)). rMSSD, root mean-square successive difference; TD, typically developing; ASD, autism spectrum disorder.

**A B**

**ADI-RRB: Linear age controlled**

**ADI-RRB: Quadratic age controlled**


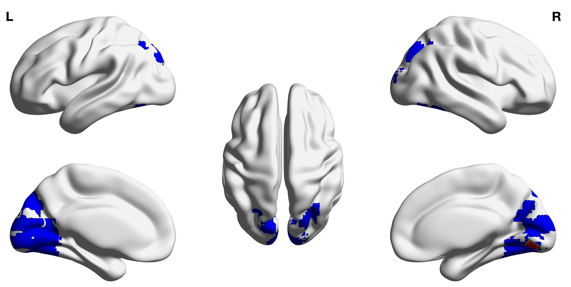

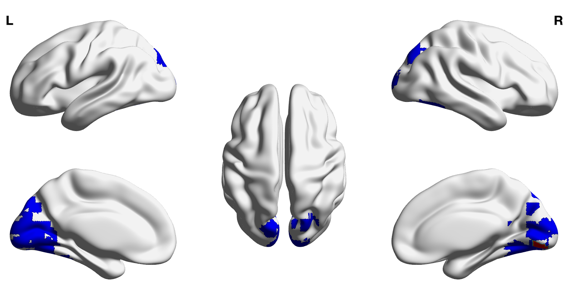


**C**

**ADI-RRB x Quadratic age interaction**


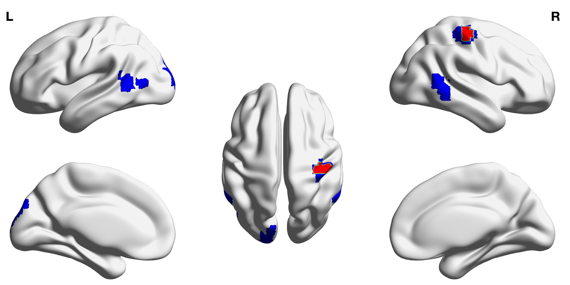


*
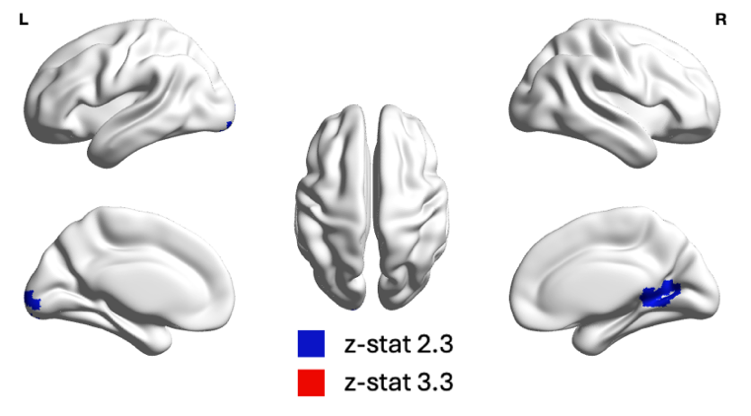
*

**Figure 2 | ADI-BSV relationship**

To examine the impact of IQ, gender, and handedness on relationship between BSV and ADI-RRB scores, we performed ﻿post-hoc regression analysis using the FSL GLM model. We conducted this analysis using a sample of 256 participants, as the remaining subjects lacked information on either gender, handedness, or ADI-RRB scores. Linear (**A**) and Quadratic Age main effect (**B**) | Brain areas showing a significant positive relationship between rMSSD and ADI-RRB scores (voxel-wise uncorrected, (z > 2.3) and cluster-wise corrected (*p* < 0.05)). Quadratic age x ADI-RRB scores interaction effect. (**C) |** Brain areas showing a significant negative relationship between rMSSD and quadratic age (voxel-wise uncorrected, (z > 2.3 & 3.3) and cluster-wise corrected (*p* < 0.05)). rMSSD, root mean-square successive difference; ADI-RRB, repetitive restricted behavior indexed by Autism Diagnostic Interview; TD, typically developing; ASD, autism spectrum disorder.
