# Supplementary material for: Age-related changes in brain signal variability in autism spectrum disorder
Source: Mol Autism. 2025 Feb 8;16:8. doi: 10.1186/s13229-024-00631-3 (PMC11806755; doi:10.1186/s13229-024-00631-3)
Supplement: Supplementary file 2 — Additional file 2: Data availability. This file contains the subject IDs obtained from the ABIDE dataset, which were utilized for our analysis. [file 13229_2024_631_MOESM2_ESM.docx]

| 28741 |
| --- |
| 28742 |
| 28743 |
| 28744 |
| 28745 |
| 28746 |
| 28747 |
| 28748 |
| 28749 |
| 28750 |
| 28751 |
| 28752 |
| 28753 |
| 28754 |
| 28755 |
| 28756 |
| 28757 |
| 28758 |
| 28759 |
| 28760 |
| 28761 |
| 28762 |
| 28763 |
| 28764 |
| 28765 |
| 28767 |
| 28768 |
| 28769 |
| 28770 |
| 28771 |
| 28772 |
| 28774 |
| 28775 |
| 28776 |
| 28777 |
| 28778 |
| 28779 |
| 28780 |
| 28783 |
| 28785 |
| 28786 |
| 28787 |
| 28788 |
| 28789 |
| 28790 |
| 28791 |
| 28792 |
| 28793 |
| 28794 |
| 28795 |
| 28796 |
| 28797 |
| 28798 |
| 28799 |
| 28800 |
| 28801 |
| 28802 |
| 28803 |
| 28805 |
| 28806 |
| 28807 |
| 28808 |
| 28809 |
| 28810 |
| 28811 |
| 28812 |
| 28813 |
| 28814 |
| 28815 |
| 28816 |
| 28817 |
| 28820 |
| 28821 |
| 28822 |
| 28824 |
| 28825 |
| 28826 |
| 28827 |
| 28828 |
| 28829 |
| 28830 |
| 28835 |
| 28836 |
| 28837 |
| 28838 |
| 28840 |
| 28841 |
| 28842 |
| 28843 |
| 28844 |
| 28845 |
| 28846 |
| 28847 |
| 28852 |
| 28853 |
| 28854 |
| 28855 |
| 28857 |
| 28858 |
| 28859 |
| 28860 |
| 28861 |
| 28862 |
| 28863 |
| 28864 |
| 28865 |
| 28866 |
| 28867 |
| 28868 |
| 28869 |
| 28870 |
| 28871 |
| 28872 |
| 28873 |
| 28874 |
| 28875 |
| 28876 |
| 28877 |
| 28878 |
| 28879 |
| 28880 |
| 28881 |
| 28882 |
| 28883 |
| 28884 |
| 28885 |
| 28886 |
| 28887 |
| 28888 |
| 28889 |
| 28890 |
| 28891 |
| 28892 |
| 28893 |
| 28894 |
| 28895 |
| 28896 |
| 28897 |
| 28898 |
| 28899 |
| 28900 |
| 28901 |
| 28902 |
| 28903 |
| 28904 |
| 28905 |
| 28906 |
| 28907 |
| 28908 |
| 28909 |
| 29057 |
| 29058 |
| 29059 |
| 29060 |
| 29063 |
| 29066 |
| 29068 |
| 29070 |
| 29071 |
| 29072 |
| 29073 |
| 29074 |
| 29075 |
| 29076 |
| 29077 |
| 29078 |
| 29080 |
| 29081 |
| 29082 |
| 29083 |
| 29084 |
| 29085 |
| 29086 |
| 29087 |
| 29088 |
| 29089 |
| 29090 |
| 29091 |
| 29092 |
| 29093 |
| 29094 |
| 29162 |
| 29177 |
| 29181 |
| 29182 |
| 29183 |
| 29186 |
| 29187 |
| 29188 |
| 29189 |
| 29191 |
| 29192 |
| 29193 |
| 29194 |
| 29195 |
| 29196 |
| 29198 |
| 29199 |
| 29200 |
| 29201 |
| 29202 |
| 29203 |
| 29204 |
| 29206 |
| 29208 |
| 29209 |
| 29210 |
| 29211 |
| 29212 |
| 29213 |
| 29214 |
| 29221 |
| 29222 |
| 29224 |
| 29225 |
| 29226 |
| 29228 |
| 29229 |
| 29230 |
| 29232 |
| 29234 |
| 29235 |
| 29236 |
| 29237 |
| 29239 |
| 29241 |
| 29243 |
| 29245 |
| 29246 |
| 29249 |
| 29250 |
| 29253 |
| 29254 |
| 29497 |
| 29498 |
| 29499 |
| 29500 |
| 29505 |
| 29512 |
| 29513 |
| 29514 |
| 29515 |
| 29516 |
| 29517 |
| 29519 |
| 29522 |
| 29523 |
| 29524 |
| 29526 |
| 29997 |
| 29998 |
| 29999 |
| 30000 |
| 30002 |
| 30003 |
| 30004 |
| 30005 |
| 30006 |
| 30007 |
| 30008 |
| 30009 |
| 30010 |
| 30011 |
| 30012 |
| 30013 |
| 30014 |
| 30015 |
| 30016 |
| 30017 |
| 30019 |
| 30020 |
| 30021 |
| 30022 |
| 30023 |
| 30024 |
| 30027 |
| 30028 |
| 30029 |
| 50182 |
| 50183 |
| 50184 |
| 50186 |
| 50187 |
| 50188 |
| 50189 |
| 50190 |
| 50191 |
| 50193 |
| 50194 |
| 50195 |
| 50196 |
| 50197 |
| 50198 |
| 50199 |
| 50200 |
| 50201 |
| 50202 |
| 50203 |
| 50204 |
| 50205 |
| 50206 |
| 50207 |
| 50208 |
| 50209 |
| 50210 |
| 50211 |
| 50212 |
| 50213 |
| 50214 |
| 50215 |
| 50216 |
| 50217 |
| 50232 |
| 50233 |
| 50234 |
| 50235 |
| 50236 |
| 50237 |
| 50238 |
| 50239 |
| 50240 |
| 50241 |
| 50243 |
| 50244 |
| 50245 |
| 50246 |
| 50247 |
| 50248 |
| 50249 |
| 50250 |
| 50251 |
| 50252 |
| 50253 |
| 50254 |
| 50255 |
| 50257 |
| 50259 |
| 50260 |
| 50261 |
| 50262 |
| 50263 |
| 50264 |
| 50265 |
| 50266 |
| 50267 |
| 50268 |
| 50269 |
| 50270 |
| 50271 |
| 50272 |
| 50273 |
| 50274 |
| 50275 |
| 50276 |
| 50277 |
| 50278 |
| 50280 |
| 50281 |
| 50282 |
| 50283 |
| 50284 |
| 50285 |
| 50287 |
| 50288 |
| 50289 |
| 50290 |
| 50291 |
| 50292 |
| 50294 |
| 50295 |
| 50296 |
| 50297 |
| 50298 |
| 50300 |
| 50301 |
| 50302 |
| 50307 |
| 50310 |
| 50314 |
| 50315 |
| 50318 |
| 50319 |
| 50320 |
| 50321 |
| 50324 |
| 50325 |
| 50326 |
| 50328 |
| 50330 |
| 50332 |
| 50333 |
| 50334 |
| 50335 |
| 50336 |
| 50337 |
| 50338 |
| 50342 |
| 50343 |
| 50344 |
| 50345 |
| 50346 |
| 50347 |
| 50348 |
| 50349 |
| 50350 |
| 50351 |
| 50352 |
| 50353 |
| 50354 |
| 50355 |
| 50356 |
| 50357 |
| 50359 |
| 50360 |
| 50361 |
| 50362 |
| 50363 |
| 50364 |
| 50365 |
| 50366 |
| 50367 |
| 50368 |
| 50369 |
| 50370 |
| 50371 |
| 50372 |
| 50373 |
| 50374 |
| 50376 |
| 50377 |
| 50378 |
| 50379 |
| 50380 |
| 50381 |
| 50382 |
| 50383 |
| 50385 |
| 50386 |
| 50387 |
| 50388 |
| 50390 |
| 50391 |
| 50397 |
| 50399 |
| 50402 |
| 50404 |
| 50405 |
| 50406 |
| 50407 |
| 50408 |
| 50410 |
| 50411 |
| 50412 |
| 50413 |
| 50414 |
| 50415 |
| 50416 |
| 50418 |
| 50419 |
| 50421 |
| 50422 |
| 50423 |
| 50424 |
| 50425 |
| 50426 |
| 50428 |
| 50433 |
| 50434 |
| 50435 |
| 50436 |
| 50438 |
| 50439 |
| 50440 |
| 50441 |
| 50442 |
| 50443 |
| 50444 |
| 50445 |
| 50446 |
| 50449 |
| 50450 |
| 50451 |
| 50452 |
| 50454 |
| 50455 |
| 50456 |
| 50457 |
| 50459 |
| 50460 |
| 50461 |
| 50462 |
| 50463 |
| 50464 |
| 50466 |
| 50467 |
| 50468 |
| 50470 |
| 50471 |
| 50472 |
| 50473 |
| 50474 |
| 50475 |
| 50476 |
| 50477 |
| 50480 |
| 50482 |
| 50483 |
| 50484 |
| 50485 |
| 50486 |
| 50488 |
| 50490 |
| 50491 |
| 50492 |
| 50493 |
| 50495 |
| 50496 |
| 50497 |
| 50498 |
| 50499 |
| 50500 |
| 50502 |
| 50504 |
| 50505 |
| 50507 |
| 50509 |
| 50511 |
| 50512 |
| 50513 |
| 50514 |
| 50515 |
| 50516 |
| 50517 |
| 50523 |
| 50524 |
| 50525 |
| 50526 |
| 50527 |
| 50528 |
| 50529 |
| 50530 |
| 50532 |
| 50551 |
| 50552 |
| 50553 |
| 50554 |
| 50555 |
| 50556 |
| 50557 |
| 50558 |
| 50559 |
| 50560 |
| 50561 |
| 50562 |
| 50563 |
| 50565 |
| 50566 |
| 50567 |
| 50568 |
| 50569 |
| 50570 |
| 50571 |
| 50572 |
| 50573 |
| 50574 |
| 50575 |
| 50576 |
| 50577 |
| 50578 |
| 50601 |
| 50602 |
| 50603 |
| 50604 |
| 50605 |
| 50606 |
| 50607 |
| 50608 |
| 50609 |
| 50610 |
| 50611 |
| 50612 |
| 50613 |
| 50614 |
| 50616 |
| 50617 |
| 50618 |
| 50619 |
| 50620 |
| 50621 |
| 50622 |
| 50623 |
| 50625 |
| 50627 |
| 50628 |
| 50953 |
| 50955 |
| 50956 |
| 50958 |
| 50959 |
| 50960 |
| 50961 |
| 50962 |
| 50964 |
| 50965 |
| 50966 |
| 50968 |
| 50970 |
| 50971 |
| 50972 |
| 50973 |
| 50974 |
| 50976 |
| 50977 |
| 50978 |
| 50979 |
| 50980 |
| 50981 |
| 50982 |
| 50983 |
| 50985 |
| 50986 |
| 50987 |
| 50988 |
| 50989 |
| 50990 |
| 50991 |
| 50992 |
| 50993 |
| 50994 |
| 50995 |
| 50996 |
| 50998 |
| 50999 |
| 51001 |
| 51002 |
| 51003 |
| 51006 |
| 51007 |
| 51008 |
| 51009 |
| 51010 |
| 51011 |
| 51014 |
| 51015 |
| 51016 |
| 51017 |
| 51018 |
| 51019 |
| 51020 |
| 51021 |
| 51023 |
| 51024 |
| 51025 |
| 51026 |
| 51027 |
| 51029 |
| 51032 |
| 51036 |
| 51041 |
| 51042 |
| 51044 |
| 51045 |
| 51046 |
| 51047 |
| 51048 |
| 51049 |
| 51050 |
| 51051 |
| 51052 |
| 51053 |
| 51055 |
| 51056 |
| 51057 |
| 51058 |
| 51059 |
| 51060 |
| 51061 |
| 51062 |
| 51063 |
| 51064 |
| 51065 |
| 51066 |
| 51067 |
| 51068 |
| 51070 |
| 51071 |
| 51072 |
| 51073 |
| 51074 |
| 51075 |
| 51076 |
| 51077 |
| 51078 |
| 51079 |
| 51080 |
| 51081 |
| 51082 |
| 51083 |
| 51085 |
| 51086 |
| 51087 |
| 51088 |
| 51090 |
| 51093 |
| 51094 |
| 51095 |
| 51096 |
| 51097 |
| 51099 |
| 51100 |
| 51101 |
| 51102 |
| 51103 |
| 51104 |
| 51105 |
| 51106 |
| 51107 |
| 51108 |
| 51109 |
| 51111 |
| 51112 |
| 51113 |
| 51114 |
| 51115 |
| 51117 |
| 51118 |
| 51119 |
| 51120 |
| 51121 |
| 51122 |
| 51125 |
| 51126 |
| 51127 |
| 51128 |
| 51129 |
| 51130 |
| 51131 |
| 51132 |
| 51133 |
| 51134 |
| 51135 |
| 51137 |
| 51138 |
| 51139 |
| 51140 |
| 51141 |
| 51142 |
| 51146 |
| 51147 |
| 51148 |
| 51149 |
| 51156 |
| 51159 |
